# Genetic Disorders of Bone or Osteodystrophies of Jaws—A Review

**DOI:** 10.1055/s-0041-1724105

**Published:** 2021-03-15

**Authors:** Sirisha Vammi, Jaya Lakshmi Bukyya, Anulekha Avinash CK, M. L. Avinash Tejasvi, Archana Pokala, Chanchala HP, Priyanka Talwade, Praveen Kumar Neela, T. K. Shyamilee, Mary Oshin, Veenila Pantala

**Affiliations:** 1 Private Practitioner, Oral Medicine and Radiology, Vishakapatnam, Andhra Pradesh, India; 2 Department of Oral Medicine and Radiology, Tirumala Institute of Dental Sciences, Nizamabad, Telangana, India; 3 Department of Prosthodontics, Kamineni Institute of Dental Sciences, Narketpally, Telangana, India; 4 Department of Oral Medicine and Radiology, Kamineni Institute of Dental Sciences, Narketpally, Telangana, India; 5 Department of Pedodontics and Preventive Dentistry, JSS Dental College, Mysore, Karnataka, India; 6 Department of Orthodontics, Kamineni Institute of Dental Sciences, Narketpally, Telangana, India; 7 Private Practitioner, MDS in Oral Pathology, Hyderabad, Telangana, India; 8 Department of Oral Pathology, Tirumala Institute of Dental Sciences, Nizamabad, Telangana, India

**Keywords:** PTH—Parathyroid hormone, CRI—Chronic renal failure, RANKL—Receptor activator of NF-B ligand, TGF—Transforming growth factor, M-CSF—Macrophage colony stimulating factor, FGFR3—Fibroblast growth factor receptor

## Abstract

Bone is a specialized form of connective tissue, which is mineralized and made up of approximately 28% type I collagen and 5% noncollagenous matrix proteins. The properties of bone are very remarkable, because it is a dynamic tissue, undergoing constant renewal in response to mechanical, nutritional, and hormonal influences. In 1978, “The International Nomenclature of Constitutional Diseases of Bone” divided bone disorders into two broad groups: osteochondrodysplasias and dysostoses. The osteochondrodysplasia group is further subdivided into two categories: dysplasias (abnormalities of bone and/or cartilage growth) and osteodystrophies (abnormalities of bone and/or cartilage texture). The dysplasias form the largest group of bone disorders, hence the loose term “skeletal dysplasia” that is often incorrectly used when referring to a condition that is in reality an osteodystrophy or dysostosis. The word “dystrophy” implies any condition of abnormal development. “Osteodystrophies,” as their name implies, are disturbances in the growth of bone. It is also known as osteodystrophia. It includes bone diseases that are neither inflammatory nor neoplastic but may be genetic, metabolic, or of unknown origin. Recent studies have shown that bone influences the activity of other organs, and the bone is also influenced by other organs and systems of the body, providing new insights and evidencing the complexity and dynamic nature of bone tissue. The 1,25-dihydroxyvitamin D3, or simply vitamin D, in association with other hormones and minerals, is responsible for mediating the intestinal absorption of calcium, which influences plasma calcium levels and bone metabolism. Diagnosis of the specific osteodystrophy type is a rather complex process and various biochemical markers and radiographic findings are used, so as to facilitate this condition. For diagnosis, we must consider the possibility of lesions related to bone metabolism altered by chronic renal failure (CRI), such as the different types of osteodystrophies, and differentiate from other possible neoplastic and/or inflammatory pathologies. It is important that the dentist must be aware of patients medical history, suffering from any systemic diseases, and identify the interference of the drugs and treatments to control them, so that we can able to perform the correct diagnosis and propose the most adequate treatment and outcomes of the individuals with bone lesions.

## Introduction

Bone is a specialized form of connective tissue that serves as both a tissue and an organ system within higher vertebrates. The adult human skeleton is composed of 80% cortical bone and 20% trabecular bone overall. 1 Therefore, its basic functions include locomotion, protection, and mineral homeostasis. Its cellular makeup includes osteoblasts, osteocytes, bone lining cells, and osteoclasts, and its matrix contains an organic and an inorganic component. 2 Bones develop during embryonic life by one of two processes. Flat bones such as the calvarium of the skull, mandible, and maxilla develop by intramembranous ossification, in which the bone forms by differentiation of mesenchymal stem cells directly into bone cells. 3 During initial bone formation, the mineralization process is complex and well regulated, and it occurs quickly once initiated. Calcium phosphate granules are found within an osteoblasts mitochondria and matrix vesicles, which are membrane-bound extracellular structures formed from the plasma membrane of osteoblasts. 4 Growth of the skeleton depends on division of cartilage cells (chondrocytes) within the growth plate. This takes place in the so-called “proliferative zone” near the end of the bone; the newly formed chondrocytes migrate downward toward the center of the bone, where they become enlarged in the hypertrophic zone. 5 During puberty, the rise in circulating concentrations of sex hormones causes cell division in the growth plate to cease. This causes the cartilage remnant to disappear, as the epiphyses fuse and longitudinal bone growth stops. 6 Bone remodeling is a highly complex process by which old bone is replaced by new bone, in a cycle comprising three phases: (1) initiation of bone resorption by osteoclasts, (2) the transition (or reversal period) from resorption to new bone formation, and (3) the bone formation by osteoblasts. Normal bone remodeling is necessary for fracture healing, skeleton adaptation to mechanical use, as well as calcium homeostasis. 7 Activation involves recruitment and activation of mononuclear monocyte-macrophage osteoclast precursors from the circulation, lifting of the endosteum that contains the lining cells off the bone surface, and fusion of multiple mononuclear cells to form multinucleated preosteoclasts. 8 Preosteoclasts bind to bone matrix via interactions between integrin receptors in their cell membranes and (arginine, glycine, and asparagine (RGD)-containing peptides in matrix proteins, to form annular sealing zones around bone-resorbing compartments beneath multinucleated osteoclasts. 9 Osteoclast formation, activation, and resorption are regulated by the ratio of receptor activator of NF-B ligand (RANKL) to osteoprotegerin (OPG), IL-1 and IL-6, colony-stimulating factor (CSF), parathyroid hormone, 1,25-dihydroxyvitamin D, and calcitonin as shown. 10

The extracellular matrix of the bone is composed of inorganic salts and organic matrix. 11 The organic matrix contains collagenous proteins (90%), predominantly type I collagen, and noncollagenous proteins including osteocalcin, osteonectin, osteopontin, fibronectin and bone sialoprotein II, bone morphogenetic proteins (BMPs), and growth factors. There are also small leucine-rich proteoglycans including decorin, biglycan, lumican, osteoaderin, and seric proteins. 12 The inorganic material of bone consists predominantly of phosphate and calcium ions; however, significant amounts of bicarbonate, sodium, potassium, citrate, magnesium, carbonate, fluorite, zinc, barium, and strontium are also present. 13 Integrins are the most common adhesion molecules involved in the interaction between bone cells and bone matrix. The most common integrins present in osteoblasts are *α* 1 *β* 1, *α* 2 *β* 1, and *α* 5 *β* 1. These proteins also play an important role in osteoblast organization on the bone surface during osteoid synthesis. 14 The interaction between osteoclasts and bone matrix is essential for osteoclast function; bone resorption occurs only when osteoclasts bind to mineralized bone surface. 15 Thus, during bone resorption osteoclasts express *α* v *β* 3 and *α* 2 *β* 1 integrins to interact with the extracellular matrix, in which the former bind to bone-enriched RGD-containing proteins such as bone sialoprotein and osteopontin, whereas *β* 1 integrins bind to collagen fibrils. 16

The local and systemic factors that regulate the bone homeostasis include autocrine and paracrine molecules such as growth factors, cytokines, and prostaglandins produced by the bone cells besides factors of the bone matrix that are released during bone resorption. 17 The systemic factors which are important to the maintenance of bone homeostasis include parathyroid hormone (PTH), calcitonin, 1,25-dihydroxyvitamin D3 (calcitriol), glucocorticoids, androgens, and estrogens. Similar to PTH, PTH-related protein (PTHrP), which also binds to PTH receptor, has also been reported to influence bone remodeling. 18 Estrogen plays crucial roles for bone tissue homeostasis; the decrease in estrogen level at menopause is the main cause of bone loss and osteoporosis. The estrogen suppresses the osteoclasts formation and activity as well as induces osteoclast apoptosis. Moreover, estrogen stimulates these bone cells to produce OPG, a decoy receptor of RANK in osteoclast, thus inhibiting osteoclastogenesis. 19

Bone mass accounts for 50 to 70% of bone strength. Bone geometry and composition are important, because larger bones are stronger than smaller bones, even with equivalent bone mineral density. The amount and proportion of trabecular and cortical bone at a given skeletal site affect bone strength independently. 20 Mutations in certain proteins may cause bone weakness (e.g., collagen defects cause decreased bone strength in osteogenesis imperfecta, impaired gamma-carboxylation of Gla proteins). 21 Bone strength can be affected by osteomalacia, fluoride therapy, or hypermineralization states. Bone microstructure affects bone strength also. Low-bone turnover leads to accumulation of microfractures. High-bone turnover, with bone resorption greater than bone formation, is the main cause of microarchitectural deterioration. 22

## Diseases of Bone

Bone is a dense calcified tissue which is specifically affected by a variety of diseases that often cause it to react in a dynamic fashion. Skeletal dysplasias are a heterogenous group of disorders, which result in disproportionate short stature. The nomenclature of these disorders remains confusing. In an attempt to develop uniformity, an international nomenclature and classification was proposed in 1969. In the 1992 revision, the classification was based on radiodiagnostic and morphological criteria. In the 1997 revision, the groups of disorders were rearranged based on current etiopathogenetic information regarding the gene and/or protein defect in these disorders. In the 2001 revision, the term dysostoses was incorporated in the nomenclature. All these revisions merely reflect the complexity of skeletal-genetic phenotypes.

## Definitions 5

**Skeletal dysplasia:** Heterogenous group of disorders, which result in disproportionate short stature.
**Osteochondrodysplasia:** This refers to abnormalities of cartilage and bone growth and development. It denotes generalized disorders of the skeletal system, encompassing multiple bones at the time of presentation.
**Dysostoses:** Refers to malformations of individuals bones, single or in combination, and does not refer to a generalized disorder of the skeleton. Many disorders that were previously referred to as dysostoses are now listed with the osteochondrodysplasias, since they are due to mutations of genes associated with dysplasias and, therefore, of a more generalized nature.
**Osteodystrophies:** Diseases of bone in which there is failure of normal development or abnormal metabolism in the bone, which is usually associated with disturbances in calcium and phosphorus metabolism and renal insufficiency such as in renal osteodystrophy.

## Etiology

1. **Disturbance in the growth of bone:** Genetic abnormalities can produce weak, thin bones, or bones that are too dense. Nutritional deficiencies, particularly of vitamin D, calcium, and phosphorus, can result in the formation of weak, poorly mineralized bone. Many hormonal disorders can also affect the skeleton. Use of glucocorticoids as medication is a common cause of bone disease. Inflammation can lead to bone loss, probably through the production of local resorbing factors by the inflammatory white cells.
2. **Disturbance in the osteogenic and osteolytic balance.**
3. **Disturbance in the deposition of hydroxyapatite crystals in the matrix of osteoid tissue.**

## Classification of Genetic Osteodystrophies 5

- Osteogenesis imperfecta.
- Achondroplasia.
- Marfan's syndrome.
- Osteopetrosis.
- Cleidocranial dysplasia.
- Hypophosphatasia.
- Cherubism.
- Crouzon syndrome.
- Treacher Collins syndrome (TCS).
- Sickle cell anemia.
- Thalassemia major.

**Osteogenesis imperfecta (brittle bones disease, Lobstein's disease) (OI):** It is a genetic disorder characterized by bones that break easily and literally means “imperfectly formed bone.” People with OI have a genetic defect that impairs the body's ability to make strong bones. It is also known as “brittle bone disease.” Severely affected patients have multiple fractures since childhood, both spontaneous and related to minimal trauma, and the most seriously affected children usually die in the neonatal period. OI is a rare clinical disease, occurring at a rate of between 1/10,000 and 1/25,000 worldwide. 23 The first scientific description of OI was given by the Swedish army surgeon Olaus Jakob Ekman who, in his thesis on “congenital osteomalacia,” described a three-generation family with hereditary bone fragility. 21 Looser 47 made the first classification of OI into congenital and tarda. Attempts were made to further classify OI, and in 1979, the “Sillence classification” was proposed which, though in an adjusted form, is still in use today. 24 Sykes 48 reported altered relation of two collagen types in pepsin digests of skin of OI patients, using a method of interrupted polyacrylamide-gel electrophoresis. Type I: autosomal dominant associated with osseous fragility (variable), adulthood hearing loss, blue sclera; type II: autosomal dominant, autosomal recessive associated with extremely severe osseous fragility, perinatally lethal; type III: autosomal recessive associated with moderate-to-severe osseous fragility, normal sclera, severe deformity of long bones and spine, variable clinical and radiographic phenotypes; type IV: autosomal dominant, autosomal recessive associated with osseous fragility, generally normal sclera, and severe deformity of long bones and spine.

**Clinical features** 24 : The majority of OI cases (possibly 85 to 90 percent) are caused by a dominant mutation in a gene coding for type 1 collagen (types I, II, III, and IV in the following list). Types V and VI do not have a type 1 collagen mutation, but the genes causing them have not yet been identified. Types VII and VIII are newly discovered forms that are inherited in a recessive manner, and the genes causing these two types have been identified.

**Type I OI:** Most common and mildest type of OI. Loose joints and muscle weakness, blue, purple, or gray tint to sclera (whites of the eyes), triangular face, possible hearing loss, often beginning in early twenties or thirties.

**Type II OI:** Most severe form of OI. Frequently causes death at birth or shortly after, because of respiratory problems, numerous fractures and severe bone deformities and blue, purple, or gray tinted sclera.

**Type III OI:** Most severe type among those who survive the neonatal period with barrel-shaped rib cage and triangular face.

**Type IV OI:** Between type I and type III OI in severity. Most cases of OI (85 to 90 percent) are caused by a dominant genetic defect. Approximately 10 to 15 percent of OI cases are the result of a recessive mutation.

**Oral manifestations:** Both dentitions are involved and demonstrate blue to brown translucence, large head size, frontal and temporal bossing, exaggerated occiput, class III malocclusion due to maxillary hypoplasia with or without mandibular hyperplasia, anterior and posterior cross bites and open bite, large number impactions, and ectopic teeth. In permanent dentition, OI patients have unerupted first and second molars.

**Radiographic features depending on the type of OI: hyperplastic callus formation.** 25 This has occasionally been reported in type V OI, especially in males and in the femur. Hyperplastic callus formation can occur either after a fracture or surgery or spontaneously, and it may mimic osteosarcoma clinically and radiographically. **Ossifications of the interosseous membrane:** these are encountered in type V OI, in the forearm or leg, and may be associated in some cases with a congenital dislocation of the radial head. **“Popcorn” calcifications** : They are more commonly seen in type III OI, in the metaphyseal and epiphyseal regions of the knee, and may contribute to femoral growth deficiency and lower leg-length discrepancy. Crowns are normal in size, but there is constriction of cervical portion of tooth, which gives the crown a bulbous appearance. Roots are short, partial or complete obliteration of the pulp chambers and root canals. Deciduous teeth appear to have large pulp chambers, but these are quickly obliterated by formation of dentin. Rarefying osteitis may be seen due to the presence of residual pulp.

**Diagnosis** 23 : It is often possible to diagnose OI, based solely on clinical features. Clinical geneticists can perform biochemical (collagen) or molecular (DNA) tests that can help confirm a diagnosis of OI in some situations. Most cases diagnosed prenatally by ultrasound are type II, with fewer case of type III. This is because types I and IV can be normal before birth, and types V to VIII are extremely rare in clinical practice. Serum calcium is normal in patients with OI, while serum 25-hydroxyvitamin D is often low. Biochemical markers for bone turnover do not provide accurate information on bone structure in OI, although a selective decrease in serum levels of carboxyterminal propeptide of type 1 collagen (PICP) has been reported.

However, other bone turnover markers such as osteocalcin, alkaline phosphatase, and aminoterminal telopeptide of type 1 collagen are useful for monitoring children with OI. The first technique used electrophoresis of type I collagen protein combined with a COLIA1/2 gene sequence test. The latter used multiplex ligation-dependent probe amplification (MLPA). They found that MLPA was the superior and more reliable method for finding genetic abnormalities.

**Management** 26 : Antisense suppression therapy aims at selectively decreasing or silencing the expression of the mutant allele, without interfering with the expression of the normal allele, and thus biochemically transforming a severe form of OI into a mild form. In the case of the structural defect of the collagen, apart from gene therapy, the other chance is molecule replacement of cells carrying the mutant gene with normal cells, essentially, with the help of bone marrow transplantation (BMT).

**Achondroplasia:** Achondroplasia is autosomal dominant trait which brings defect in cartilaginous bone formation. It is a type of dwarfism with characteristics of short stature and short limbs. The term “achondroplasia” was first used in 1878 by Dr. Joseph Jules Marie Parrot (French physician). The prevalence of this disorder is 1 in 15000. Achondroplasia literally means failure of endochondral bone formation. 27 Achondroplasia is part of a spectrum of disorders caused by different mutations in FGFR3, which includes hypochondroplasia (OMIM 146000), severe achondroplasia with developmental delay and acanthosis nigricans (SADDAN), and thanatophoric dysplasia, of which two types can be distinguished by radiograph and molecular analysis (thanatophoric dysplasia I [OMIM 187601] and thanatophoric dysplasia II [OMIM 187601]). 28 Most of the **clinical features** of achondroplasia are either direct or indirect consequences of increased fibroblast growth factor receptor 3 (FGFR3) signaling on endochondral bone growth. For instance, neurological manifestations in infants and adults with achondroplasia are typically the result of diminished growth of the base of the skull and vertebral pedicles, dental crowding due to reduced growth of the midface, and hearing loss resulting from recurrent middle ear infection. The combination of joint hypermobility and hypotonia means that many infants will seem particularly “floppy.” **Radiographic features:** typical features include:

(A) Squared iliac wings with horizontal trident-shaped acetabular roof, narrow sciatic notches, decreased interpedicular distance caudally, and proximal femoral translucency.
(B) Short metacarpals and bullet-shaped phalanges with “trident” hand configuration.
(C) Bullet-shaped vertebral bodies with short pedicles and posterior scalloping.
(D) Midface hypoplasia and frontal bossing.

**Management** 29 : The use of the FGFR3 kinase inhibitors is that FGFR3′s kinase activity is critical for its activation and signal output; chemical inhibition of its kinase activity should block its inhibitory output and restore bone growth. Trastuzumab (trade name Herceptin) is a humanized monoclonal antibody to the extracellular domain of the HER2/neu (ErbB2), a transmembrane receptor overexpressed on the surface of approximately 15–30% of breast cancer cells.

**Marfan syndrome (Marfan–Achard syndrome, arachnodactyly):** Marfan syndrome (MFS) is an autosomal dominant genetic disorder, with a prevalence of 1:5000. French pediatrician Antonin Marfan in 1896 first reported this syndrome as arachnodactyly; clinical features included abnormally long, slender or spidery fingers and toes. Currently, MFS is classified as a disorder affecting the connective tissues. In 1991, the fibrillin-1 gene (FBN1), which encodes a 350 kDa glycoprotein that was found abundantly in the extracellular matrix (ECM), was implicated in MFS. Fibrillin-1 together with other ECM proteins form thread-like microfibrils, which provide elasticity and structural support to tissues. FBN1 mutations lead to degeneration of microfibril architecture and subsequent loss of ECM integrity. Following recent implication of transforming growth factor β (TGF-β) in the etiology of MFS, that FBN1 in disease pathology has expanded from mere defective structural support to regulatory dysfunction in cellular signaling: The regulatory role of FBN1, and the implication of increased TGF-β signaling in the development of some manifestations of the disease such as impaired pulmonary alveolar septation or myxomatous thickening of mitral valve. 30 **Clinical manifestation:** Tall slender stature with relatively long legs and arms, long hands, and long fingers. The skeleton typically displays multiple deformities including arachnodactyly, dolichostenomelia (long limbs relative to trunk length), and thoracolumbar scoliosis. Hyperextensibility of joints with habitual dislocations, kyphosis and flat feet. A narrow, high-arched palatal vault with crowding of teeth is very prevalent. Bifid uvula, dolichocephaly, retrognathia, micrognathia and convex profile, and malocclusion. Multiple odontogenic cysts of the maxilla and mandible have occasionally been reported. Temperomandibular dysarthrosis may be present. Greater risk of dental caries and gingival inflammation, root deformity, abnormal pulp shape, pulpal calcifications, and impacted teeth.

**The radiological features:** Skull radiographs (AP and lateral) may demonstrate a high-arched palate, increased skull height and an enlarged frontal sinus. **Diagnosis** 31 : Major diagnostic criteria include enlarged aorta, tear in the aorta, dislocation of the lens, family history of the syndrome, at least four skeletal problems such as flat feet or curved spine, and dural ectasia (enlargement of the lining that surrounds part of the spinal cord), while minor criteria include short sightedness (myopia), unexplained stretch marks, and loose joints. Transesophageal echocardiography and MRI are preferred over contrast aortography for diagnosing aortic dissection in pregnant patients with MFS.

**Management:** Management is most effectively accomplished through the coordinated input of a multidisciplinary team, including a geneticist, cardiologist, ophthalmologist, orthopedist, and a cardiothoracic specialist.

**Osteopetrosis (marble bone disease, Albers–Schonberg disease):** Osteopetrosis is a group of genetic diseases characterized by increased bone mass and density due to a failure in bone resorption. The term osteopetrosis is derived from the Greek “osteo” meaning bone and “petros” meaning stone. Osteopetrosis is variably referred to as “marble bone disease” and “Albers–Schönberg disease,” after the German radiologist was credited with the first description of the condition in 1904. 32 Two major forms autosomal dominant osteopetrosis (ADO), autosomal recessive osteopetrosis (ARO) can be distinguished on the basis of their mode of inheritance: autosomal dominant osteopetrosis (ADO, formerly known as Albers–Schönberg disease) is usually considered an adult-onset, more benign form, autosomal recessive osteopetrosis (ARO) that is also termed malignant, and infantile osteopetrosis, presenting soon after birth, which is often severe and leads to death if left untreated. The form of osteopetrosis caused by hypomorphic mutations in the *IKBKG* gene on the X chromosome; the bone pathology arising from cathepsin K mutations, which is termed pycnodysostosis in humans; and the type of osteopetrosis present in patients with leucocyte adhesion deficiency III, which is caused by mutations in the *KINDLIN3* gene. 33 **Clinical features** 34 : The more severe forms tend to have autosomal recessive inheritance, while the mildest forms are observed in adults and are inherited in an autosomal-dominant manner. More severe, recessive form of osteopetrosis is seen in infants and young children. More benign, dominant form appears later. Severe form is invariably fatal early in life. Patient experiences progressive loss of the bone marrow and its cellular products and a severe increase in bone density. Narrowing of bony canals results in hydrocephalus, blindness, deafness, vestibular nerve dysfunction, and facial nerve paralysis. Medullary spaces of jaws are reduced, leading to development of osteomyelitis, Enamel hypoplasia, microscopic dentinal defects, arrested root development, teeth prone to dental caries, and retardation of tooth eruption due to sclerosis of bone. **Radiographic features of the skull:** The typical “open mouth outline” facial appearance is due to frontal bossing, micrognathia, loss of the mandibular angle, and dental anomalies including persistence of deciduous teeth, resulting in double row of teeth. Increased radiopacity of the jaws may be so great that the radiographic image may fail to reveal any internal structure. Roots of the teeth may not be apparent. Normal eruption pattern of the primary and secondary dentition may be delayed as a result of bone density or ankylosis. Lamina dura and cortical borders appear thicker than normal. **Diagnosis** 35 : The mainstay of diagnosis is clinical and largely depends on the radiographic appearance of the skeleton. The classic radiological features of osteopetrosis comprise the following: diffuse sclerosis affecting the skull, spine, pelvis, and appendicular bones; bone modelling defects at the metaphyses of long bones such as funnel-like appearance (“Erlenmeyer flask” deformity), and characteristic lucent bands. In the absence of typical radiographic findings, raised concentrations of the creatine kinase BB isoenzyme and tartrate-resistant acid phosphatase (TRAP) can be helpful in making the diagnosis of ADO. **Management:** Dental problems such as delayed tooth eruption, ankylosis, abscesses, cysts, and fistulas are common. Therefore, routine dental surveillance and maintenance of oral hygiene form an integral part of management and play an important role in preventing more severe complications such as osteomyelitis of the mandible.

**Cleidocranial dysplasia (cleidocranial dysostosis)** 36 : The term cleidocranial dysplasia (CCD; OMIM 119600) is derived from the ancient Greek words cleido (collar bone), kranion (head), and dysplasia (abnormal formation). This rare hereditary skeletal disorder, which is also known as Scheuthauer–Marie–Sainton syndrome or cleidocranial dysostosis, is characterized by abnormal skeletal and dental development. The prevalence of CCD is an estimated one per million and does not differ by race or by gender. In most cases, the disorder is an inherited autosomal dominant trait. Mutations in runt-related transcription factor 2 gene ( *RUNX2* , OMIM #600211) have been associated with the molecular basis of this disorder. *RUNX2* codifies for a core-binding transcription factor protein (CBFA1 or RUNX2), which plays an important role in the differentiation of osteoblasts, chondrocyte maturation, and regulation of bone metabolism. **Clinical features:** It affects men and women with equal frequency. CCD primarily affects the skull, clavicles, and dentition. Shorter stature than unaffected relatives but not short enough to be considered a form of dwarfism. Face appears small in contrast to the cranium because of hypoplasia of the maxilla, a brachycephalic skull (reduced anteroposterior dimension with increased skull width), and the presence of frontal and parietal bossing. Paranasal sinuses→ underdeveloped. bridge of the nose→ broad and depressed, with hypertelorism (excessive distance between the eyes). Complete absence (aplasia) or reduced size (hypoplasia) of the clavicles allows excessive mobility of the shoulder girdle.

**Radiographic features:** Characteristic skull findings are brachycephaly, delayed or failed closure of the fontanels, open skull sutures, and multiple Wormian bones (small, irregular bones in the sutures of the skull that are formed by secondary centers of ossification in the suture lines). The mandible is characterized by a narrow ascending ramus with nearly parallel anterior and posterior borders and by an abnormally slender and pointed coronoid process, with an abnormally distal curvature. Imaging by cone-beam computed tomography (CBCT) is now routinely used for three-dimensional (3D) dentition, which reduces guesswork and enables better anatomical localization of supernumerary and impacted teeth. **Management:** Radiological and clinical approaches are the most important and consistent signs and symptoms to confirm diagnosis; therefore, patients with CCD should be carefully evaluated by a dental multispecialist team on account of the complexity of the treatment foreseen.

**Hypophosphatasia:** It is a rare, inherited form of rickets or osteomalacia due to mutation (s) of the ALPL gene, resulting in reduced activity of tissue nonspecific alkaline phosphatase (TNSALP), which is marked by a low-serum alkaline phosphatase (hypophosphatasemia). Hypophosphatasia (HPP; OMIM #241500, 241510, 146300), which was first reported by the Canadian pediatrician John Campbell Rathbun in 1948, is a systemic bone disease caused by the deficiency of tissue-nonspecific alkaline phosphatase (TNAP). 37 Mutations resulting in reduced activity of TNSALP are associated with abnormal bone mineralization. In HPP, accumulation of three substrates of TNSALP occurs as a result of the genetic defect, leading to a deficiency in TNSALP activity. These three substrates are as follows: PPi, an inhibitor of hydroxyapatite crystal formation, resulting in hypomineralization of bone; pyridoxal-5′-phosphate (PLP), the major circulating form of vitamin B6; and phosphoethanolamine (PEA), a degradation product of cell surface phosphatidyl-inositol-glycan stabilizers. In HPP, hydroxyapatite crystals form normally in matrix vesicles during phase 1 of skeletal mineralization. However, excess PPi inhibits their growth after rupture of matrix vesicles during phase 2, causing rickets or osteomalacia. A compromise of phase 2 hydroxyapatite crystal formation beyond the matrix vesicle is the hallmark of HPP. Within central nervous system (CNS) cells, PLP serves as an important cofactor for several reactions including formation of an inhibitory neurotransmitter known as gamma-aminobutyric acid (GABA). A balance of inhibitor and excitatory neurotransmitters is important in the CNS. In HPP, decreased TNSALP activity leads to defective dephosphorylation of PLP to PL, resulting in accumulation of serum PLP. However, there is CNS deficiency of PLP. Reduced CNS PLP results in decreased GABA and inhibitory neurotransmitter activity, unimpeded excitatory neurotransmitter activity, and seizures. 38 **Clinical features:** About 85% of these children show premature loss of the primary teeth, particularly the incisors, and delayed eruption of the permanent dentition. Dental signs are first clinical signs of hypophosphatasia and the only sign of odontohypophosphatasia. **Radiographic features:** Generalized radiolucency of the mandible and maxilla is evident, cortical bone and lamina dura are thin, alveolar bone is poorly calcified and may appear deficient, both primary and permanent teeth have a thin enamel layer and large pulp chambers and root canals, and teeth may also be hypoplastic and may be lost prematurely. **Management:** Involves medical therapy of abnormal mineral biochemistry and, most recently, recombinant enzyme replacement to promote mineralization. Bisphosphonates are mineralization inhibitors and PPi analogs typically not used in the treatment of HPP, as they are associated with atypical fractures and can exacerbate underlying osteomalacia. Recombinant parathyroid hormone is an anabolic agent that stimulates osteoblasts and increases ALP in addition to decreasing pain, radiographic improvement of fracture healing, and improvement in bone turnover markers in adults with HPP. Asfotase alfa is a subcutaneously injected fusion protein that consists of TNSALP, the Fc fragment of immunoglobin G1, and a deca-aspartate motif for mineral targeting. 39

**Cherubism:** “Bone dystrophies paint queer and irregular pictures throughout the skeleton and have been reported in most bones.” W.A. Jones begins his 1950 review, where he proposed the name “cherubism” for the multilocular cystic disease of the jaws that he had first described 17 years earlier. The term “cherubism” was coined because “the full round cheeks and the upward cast of the eyes give the children a peculiarly” cherubic appearance. Cherubism is defined by the appearance of symmetrical, multilocular, expansile radiolucent lesions of the mandible and/or the maxilla that typically first appear at the age of 2 to 7 years. Swelling of submandibular lymph nodes in the early stages contributes to the fullness of the face. As the soft fibrous dysplastic tissue in the lesions expands, the protuberant masses can infiltrate the orbital floor and cause the characteristic upward tilting of the eyes, exposing the sclera below the iris. 40 Cherubism is classically transmitted as an autosomal dominant trait, but there are indications that a recessive form may also exist. Gene map and protein structure of human SH3BP2 indicate mutations in the canonical cherubism mutation interval (amino acids 415–420) and mutations reported in the pleckstrin homology (PH) domain. **Clinical features:** Painless symmetric enlargement of posterior region of mandible, with expansion of alveolar process and ascending ramus. With maxillary disease, involvement of the orbital floor and the anterior wall of the antrum occurs. Eyes turned to heaven. **Radiographic features:** Radiographically, cherubism is characterized by expansive radiolucent, generally multiloculated lesions, clearly delimited by cortical bone and distributed bilaterally in the posterior quadrants of the mandible and/or maxilla. Radiographically identified multilocular radiolucent lesions mainly affect the body and ascending ramus of the mandible. **Diagnosis:** Mineral metabolism is normal in patients with cherubism, and serum levels of calcium, parathyroid hormone (PTH), parathyroid hormone-related peptide (PTHrP), calcitonin, and alkaline phosphatase (ALP) are typically within normal range. Biochemical analysis can differentiate cherubism from hyperparathyroidism, particularly in patients with brown tumors (epulis) of the jaw or patients with the hyperparathyroidism-jaw tumor (HPT-JT) syndrome with mutations in the HRPT2 gene encoding parafibromin. **Management:** Mild forms of cherubism without facial dysmorphology, dental, and ocular involvement may not require treatment, as they are expected to regress spontaneously after puberty. However, radiation therapy is contraindicated in this benign condition because of the potential for long-term adverse consequences such as retardation of jaw growth, osteoradionecrosis, and increased incidence of induced malignancy. 41

**Crouzon syndrome: (craniofacial dysostosis type 1 [CFD1]; Crouzon craniofacial dysostosis; Crouzon disease).** 42 The term “Crouzon syndrome” describes an autosomal disease, which results from hereditary mutations identified in specific genes in the human DNA chain. The molecular deformities most customarily occur in FGFR2 gene and, in rare instances, in the FGFR3 gene. Moreover, recent studies have identified mutations in FGFR1, MSX2, TWIST1, EFnB1, NELL1, GLI3, and TCF12 genes. These mutations result in premature synostosis of several sutures of the craniofacial complex. Specifically, the fusion of the sagittal and coronal sutures begins, in most of the cases, during the first year of life, leading to the growth inhibition at the affected sutures and compensative growth at other sutures. It is one of the most frequent craniosynostosis syndromes, with estimated incidence of 1:60000 live births. Craniosynostosis, which can be suspected during antenatal stage via ultrasound scan otherwise, is often detected at birth from its classic crouzonoid features of the newborn. **Clinical features:** Brachycephaly (short skull front to back), hypertelorism (increased distance between eyes), orbital proptosis (protruding eyes), patients may become blind as a result of early suture closure and increased intracranial pressure, nose appears prominent and pointed because maxilla is narrow and short in vertical and anteroposterior dimension, anterior nasal spine is hypoplastic and retruded, failing to provide support to soft tissue of nose, palatal vault is high, and maxillary arch is narrow and retruded, resulting in crowding of teeth. **Radiographic features:** Lack of growth in an anteroposterior direction at cranial base results in maxillary hypoplasia. Maxillary hypoplasia contributes to orbital proptosis, because maxilla forms part of inferior orbital rim, and if severely hypoplastic fails to support orbital contents adequately. It is associated with class III malocclusion. **Diagnosis:** Craniosynostosis can be suspected prenatally, based on indirect signs visualized using two- (2D) or 3D ultrasound, MRI, or CT scan. **Management:** The complication and stability of the result depend on the complexity of the case, age of the patient, and type and number of the surgical technique. Complications of surgical interventions include mortality, cerebrospinal fluid (CSF) leak, intraoperative bleeding, wound infection, postoperative visual loss, distraction device failure and relapse, among others.

**TCS** 43 : (TCS, OMIM number 154500) is an autosomal dominant disorder of craniofacial morphogenesis. Also known as mandibulofacial dysostosis and Franceschetti–Zwahlen–Klein syndrome, TCS occurs with an estimated incidence of 1/50 000 live births. Genetic, physical, and transcript mapping techniques previously identified the gene mutated in TCS, designated TCOF1, which was found to encode a low complexity, serine/alanine-rich, nucleolar phosphoprotein termed treacle gene. Deletions which range in size from 1 to 40 nucleotides are by far the most common. TCS is thought to be genetically homogeneous because all the multigenerational families analyzed to date exhibit linkage to the human chromosome 5q32 locus. **Clinical features:** Underdevelopment or absence of zygomatic bones result in small, narrow face, downward slanting of palpebral fissures (antimongoloid palpebral fissures with coloboma of outer portion of lower eyelids with deficiency of eyelashes medial to coloboma), malformation of external ears, absence of external auditory canal, hypoplasia or atresia auditory canal and ossicles of middle ear, which may result in partial or complete deafness, underdevelopment of mandible, resulting in downturned mouth, occasional facial clefts resulting in macrostomia, hypoplasia of the mandible, and a steep mandibular angle resulting in angle class II anterior open bite malocclusion with prominent antegonial notch. Characteristic face: Birdlike or fishlike. **Radiographic features:** Hypoplastic mandible with prominent antegonial notch and a steep mandibular angle, short ramus, condyles positioned posteriorly and inferiorly, maxillary sinuses absent or underdeveloped. **Diagnosis:** TCS can be detected using prenatal screening ultrasound. Usually, it is difficult to get a satisfactory view of facial structures until after 30 weeks. Amniocentesis may be performed to identify the mutation and rule out other facial dysostoses such as Goldenhar or Nager syndromes, which can have similar appearance on ultrasound. **Management:** Surgical intervention, including bilateral distraction osteogenesis of the mandible, may improve the osseous defects.

**Sickle-cell anemia:** Sickle-cell disease or sickle-cell anemia (or depanocytosis) is a lifelong blood disorder characterized by red blood cells that assume an abnormal, rigid, sickle shape. Sickling decreases the cells' flexibility and results in a risk of various complications. The sickling occurs because of a mutation in the hemoglobin gene. Sickle cell anemia is caused by a point mutation in the β-globin chain of hemoglobin, causing the amino acid glutamic acid to be replaced with the hydrophobic amino acid valine at the sixth position. Healthy red blood cells typically live 90 to 120 days, but sickle cells only survive 10 to 20 days according to Emedicine. **Classification:** Sickle-cell anemia is the name of a specific form of sickle-cell disease in which there is homozygosity for the mutation that causes HbS. Sickle-cell anemia, is also referred to as “HbSS,” “SS disease,” and “hemoglobin S.” In heterozygous people, only one sickle gene and one normal adult hemoglobin gene, it is referred to as “HbAS” or sickle-cell trait. Other rarer forms disease include sickle hemoglobin C disease (HbSC), sickle β-plus thalassemia (Hbs/B) and sickle β-zero thalassemia (HbS/P). 44 **Clinical features:** Common in females, clinically manifest before the age of 30 years, patient is left weak, short of breath, and easily fatigued, pain in the joints, limbs, and abdomen, nausea and vomiting is common, systolic murmur and cardiomegaly may occur, packing of red blood cells in peripheral vessels with erythrostasis and subsequent local tissue anoxia, sickle-cell crisis. mucosal pallor, delayed eruption, discolored and depapillated tongue, periodontitis, malocclusion, dental caries, high-arched palate, vaso-occlusive events affecting the oral cavity and causing pain, and paresthesia or swelling are common oral manifestations. **Radiographic features:** Perpendicular trabeculae radiating outward from the inner table, giving a “hair-on-end” appearance, ground-glass appearance of the skull, generalized osteoporosis, trabeculae giving a stepladder-like effect between the roots of the teeth, lamina dura is not affected, thinning of the inferior cortex of mandible, increased radiolucency representing osteoporosis, radiopacities representing healed infarcts, larger marrow spaces, calcified pulp canals, hypercementosis, and enamel hypoplasia.

**Diagnosis:** In HbSS, the fall blood count (FBC) reveals hemoglobin levels in the range of 6 to 8 g/dl with a high reticulocyte count. In other forms of sickle-cell disease, Hb levels tend to be higher. A blood film may show features of hyposplenism (target cells and Howell–Jolly bodies). Sickling of the red blood cells, on a blood film, can be induced by the addition of sodium metabisulfite. The presence of sickle hemoglobin can also be demonstrated with the “sickle solubility test.” **Management:** The management of sickle-cell anemia is in two phases: quiescent stage and crisis stage. Sickle-cell patients have increased metabolic rate and protein turnover, which is balanced by high calorie intake. Folic acid requirements are increased by hemolysis above normal levels of 50 g/day, and as much as 500 mg daily has been necessary to reverse established megaloblastic change. 45

**Thalassemia (Cooley's anemia, Mediterranean anemia, erythroblastic anemia)** 44 : The name thalassemia is derived from the combination of two Greek words: *Thalassa* meaning the sea (Cooley et al, 1925, 1927; Bradford and Dye, 49 1936), that is the Mediterranean, and anemia (“weak blood”). Thalassemia is a genetic blood disorder inherited from a person's parents which can result in the abnormal formation of hemoglobin. There are two main types, α and β thalassemia. The severity of α and β thalassemia depends on how many of four genes for α or two genes for β globin are missing. Thalassemia is widespread throughout the Mediterranean region, Africa, the Middle East, the Indian subcontinent, and South-East Asia. Causes of anemia in α thalassemia is summarized with the help of four components: 1) low hemoglobin HbA, leading to reduced or absent synthesis of α globin; 2) production of two abnormal hemoglobin Hb Bart's (gamma globin tetramers) and HbH (β globin tetramers) because of insufficient numbers of α globin chain; 3) accumulation of excess unmatched non-α globin chains within red blood cells leading to intracorpuscular hemolysis, and 4) ineffective erythropoiesis. A quantitative reduction of structurally normal β globin chains result in β thalassemia. They are caused by mutations that nearly all affect the β globin locus and are extremely heterogeneous. Almost every possible defect affecting gene expression at transcription or posttranscriptional level, including translation, have been identified in β thalassemia. Anemia in β thalassemia occurs in the presence of two phenomena: the first one is infective erythropoiesis and the second is the destruction of circulating red blood cells (hemolysis). The main difference in pathophysiology between α and β thalassemia is that the excess β or gamma globin chains in the α thalassemia can form partially soluble but ineffective hemoglobin homotetramers. **Clinical manifestations:** Three main forms have been described: thalassemia major, thalassemia intermediate, and thalassemia minor. **Beta thalassemia minor:** Have only mild microcytic, hypochromic anemia. **Beta thalassemia intermediate:** Have mild-to-moderate anemia. In β thalassemia intermediate, the patients have Hb of much below 7 or 8 g/dl. **Beta thalassemia major (also called Cooley's anemia):** severe form of thalassemia. Symptoms appears in first 2 years of life. Progressive enlargement of the abdomen due to splenomegaly and hepatomegaly. Puffiness of the eyelid and a tendency of a Mongoloid slant of the eyes are common presenting symptoms. Malar prominence, frontal bossing, saddle nose and upper lip is retracted, reduced posterior facial height and increased anterior facial proportions—chipmunk faces. High-caries index due to poor oral hygiene, mucosal pallor, atrophic glossitis due to decreased hemoglobin levels, severe gingivitis, inflammation of salivary glands due to iron deposits, dark-colored gingiva due to high ferritin levels in blood, bimaxillary protrusion, mandibular atrophy, increased overjet, and anterior open bite resulting in class II malocclusion. **Radiological features:** Striations of the calvarium were observed. This is called “hair-on-end” effect, which is caused by alignment of diploic trabeculae in a position perpendicular to tables and absence of the thinned outer table because of radiologic burnout. Pituitary fossa is altered. It is characterized by lobular enlargement of pituitary fossa in the anterior region. It may be associated with stunted growth in these patients. Osteoporosis, prominent maxilla, thinning of the cortex, trabecular pattern—salt and pepper appearance, marrow space enlargement with pronounced trabeculae found in the anterior regions of the mandible and maxilla, thinning of the lamina dura and crypts of developing teeth, roots of the teeth appear short and spike-shaped, and taurodontism. **Management:** Patients with thalassemia minor require no treatment except for iron supplementation when iron deficiency is confirmed. Patients with thalassemia major require regular blood transfusion to maintain their hemoglobin levels, in order to prevent growth impairment, organ damage, and bone deformities. Iron chelation to prevent iron overload complications such as cardiomyopathy and liver cirrhosis. Iron chelators—deferoxamine, deferasirox and deferiprone. A commonly used agent is oral dispersible deferasirox. Bone marrow transplantation is the only available cure for thalassemia. 46

## Conclusion

The knowledge of the structural, molecular, and functional biology of bone is essential for the better comprehension of this tissue as a multicellular unit and a dynamic structure that can also act as an endocrine tissue, a function still poorly understood. In vitro and in vivo studies have demonstrated that bone cells respond to different factors and molecules, contributing to the better understanding of bone cells plasticity. Additionally, bone matrix integrins-dependent bone cells' interactions are essential for bone formation and resorption. Studies have addressed the importance of the lacunocanalicular system and the pericellular fluid, by which osteocytes act as mechanosensors, for the adaptation of bone to mechanical forces. The incidence of osteodystrophies continues to rise worldwide and, as a consequence, increasing numbers of individuals with such disease will probably continue to require oral health care. In such a scenario, the dentists are frequently encountered with patients who have oral manifestations of bone lesions. So, the systemic evaluation of the patient should be done before diagnosing the oral lesions. Also, we can avoid the potential complications that may arise when we treat such patients and provide them good oral hygiene. Hence, we conclude that oral manifestations of systemic diseases are more frequent, and as dentists, we have a vital role in treating them accordingly.
